# Control of Vertebrate Skeletal Mineralization by Polyphosphates

**DOI:** 10.1371/journal.pone.0005634

**Published:** 2009-05-20

**Authors:** Sidney Omelon, John Georgiou, Zachary J. Henneman, Lisa M. Wise, Balram Sukhu, Tanya Hunt, Chrystia Wynnyckyj, Douglas Holmyard, Ryszard Bielecki, Marc D. Grynpas

**Affiliations:** 1 Samuel Lunenfeld Research Institute, Mt. Sinai Hospital, Toronto, Canada; 2 Department of Chemistry, University at Buffalo, Buffalo, New York, United States of America; 3 Department of Materials Science and Engineering, University of Toronto, Toronto, Canada; 4 Department of Pathology and Laboratory Medicine, Mt. Sinai Hospital, Toronto, Canada; Illinois Institute of Technology, United States of America

## Abstract

**Background:**

Skeletons are formed in a wide variety of shapes, sizes, and compositions of organic and mineral components. Many invertebrate skeletons are constructed from carbonate or silicate minerals, whereas vertebrate skeletons are instead composed of a calcium phosphate mineral known as apatite. No one yet knows why the dynamic vertebrate skeleton, which is continually rebuilt, repaired, and resorbed during growth and normal remodeling, is composed of apatite. Nor is the control of bone and calcifying cartilage mineralization well understood, though it is thought to be associated with phosphate-cleaving proteins. Researchers have assumed that skeletal mineralization is also associated with non-crystalline, calcium- and phosphate-containing electron-dense granules that have been detected in vertebrate skeletal tissue prepared under non-aqueous conditions. Again, however, the role of these granules remains poorly understood. Here, we review bone and growth plate mineralization before showing that polymers of phosphate ions (polyphosphates: (PO_3_^−^)_n_) are co-located with mineralizing cartilage and resorbing bone. We propose that the electron-dense granules contain polyphosphates, and explain how these polyphosphates may play an important role in apatite biomineralization.

**Principal Findings/Methodology:**

The enzymatic formation (condensation) and destruction (hydrolytic degradation) of polyphosphates offers a simple mechanism for enzymatic control of phosphate accumulation and the relative saturation of apatite. Under circumstances in which apatite mineral formation is undesirable, such as within cartilage tissue or during bone resorption, the production of polyphosphates reduces the free orthophosphate (PO_4_^3−^) concentration while permitting the accumulation of a high total PO_4_^3−^ concentration. Sequestering calcium into amorphous calcium polyphosphate complexes can reduce the concentration of free calcium. The resulting reduction of both free PO_4_^3−^ and free calcium lowers the relative apatite saturation, preventing formation of apatite crystals. Identified *in situ* within resorbing bone and mineralizing cartilage by the fluorescent reporter DAPI (4′,6-diamidino-2-phenylindole), polyphosphate formation prevents apatite crystal precipitation while accumulating high local concentrations of total calcium and phosphate. When mineralization is required, tissue non-specific alkaline phosphatase, an enzyme associated with skeletal and cartilage mineralization, cleaves orthophosphates from polyphosphates. The hydrolytic degradation of polyphosphates in the calcium-polyphosphate complex increases orthophosphate and calcium concentrations and thereby favors apatite mineral formation. The correlation of alkaline phosphatase with this process may be explained by the destruction of polyphosphates in calcifying cartilage and areas of bone formation.

**Conclusions/Significance:**

We hypothesize that polyphosphate formation and hydrolytic degradation constitute a simple mechanism for phosphate accumulation and enzymatic control of biological apatite saturation. This enzymatic control of calcified tissue mineralization may have permitted the development of a phosphate-based, mineralized endoskeleton that can be continually remodeled.

## Introduction

Biomineralization processes provide fascinating, complex, and solid structures for many life forms [1]. Numerous invertebrates manipulate carbonate chemistry to control the crystallization of different calcium carbonate mineral polymorphs such as aragonite or calcite within organic matrices. These remarkable structures boast excellent material properties that provide advantages in both protection and predation. Other marine organisms, such as sponges and diatoms, biomineralize intricate silicate structures by controlling the nucleation and condensation of silicate ions.

Mineralized vertebrate skeletons instead contain crystals of apatite, a calcium- and phosphate-based mineral. In this section, we will first outline apatite, skeletal remodeling, and mineralization. We will then introduce polyphosphates and amorphous, electron-dense granules that contain calcium and phosphate and have been identified in skeletal tissue and mitochondria prepared with non-aqueous methods. Next, we will summarize tissue-nonspecific alkaline phosphatase as it relates to apatite mineralization. Thereafter, we will show how all these topics can be tied together by explaining the relationship between polyphosphates and biological apatites.

We propose that the vertebrate skeleton contains apatite mineral because apatite saturation can be enzymatically controlled by the formation and destruction of polyphosphate (polyP) ions. Calcium-polyP complexes serve as bioavailable stores of high concentrations of calcium and orthophosphate ((PO_4_)^3−^∶Pi) when apatite formation is not desired. When its mineralization is required, however, tissue-nonspecific alkaline phosphatase produces orthophosphate from polyP, concurrently releasing any calcium previously sequestered by the polyphosphate, and increasing apatite saturation.

The abbreviations in this manuscript include polyphosphate (polyP), orthophosphate (Pi), hydroxyapatite (HAP), relative saturation with respect to HAP (σ_HAP_), alkaline phosphatase (ALP), and tissue-nonspecific alkaline phosphatase (TNAP).

### Vertebrate bone - why apatite?

The vertebrate skeleton is predominately composed of bone, a mineralized tissue that is a composite of type-I collagen, non-collagenous proteins, and apatite (Ca_10_(PO_4_)_6_X_2_, X = mostly F or OH) crystals [1]. No explanations yet exist [2] for why apatite is the mineral component of choice for the vertebrate skeleton [3], [4]. In vertebrates, apatite crystals contain mostly calcium, phosphate, and carbonate [5] ions, and are on the order of tens of Ångstroms in size [6]. Biological apatites are generally not perfectly ordered on an atomic scale—they are considered poorly crystalline [7] and highly substituted with cations such as magnesium, strontium, and sodium; with anions such as fluoride; and with the polyatomic anions carbonate and hydroxyl [8]. Apatite is the only calcium-phosphate mineral phase that is stable at both a neutral and basic pH [9]. The high functionality and metabolic activity of the vertebrate skeleton suggest that apatite offers an advantage to the vertebrate organism that other minerals do not.

### Vertebrate skeletal metabolism and mineralization

Unlike invertebrate skeletons or protective shells, the vertebrate skeleton must satisfy a wide range of demands, including structural integrity, metabolic activity, growth, and continual repair of wear and damage caused by locomotion and/or trauma. These demands require that vertebrates continually resorb and rebuild their mineralized skeleton. “Remodeling” is the term used to describe this controlled destruction (resorption) and rebuilding. Newly formed bone (called osteoid) is a largely unmineralized collagenous matrix.

Mineralization of new, unmineralized skeletal tissue generally falls into two classes: intramembranous and endochondral ossification. Intramembranous ossification refers to the mineralization of newly formed osteoid. Endochondral ossification occurs in the expanding growth plate, a dynamic region of the young skeleton located beneath the soft articular cartilage that caps the ends of the growing long bones, and above the mineralized bone itself. These bones grow along their vertical axis through the progressive expansion of the epiphyseal (growth) plate. Within the active growth plates, the bone elongates as new cartilage forms on its ends. Older cartilage beneath that new-formed cartilage mineralizes with apatite; it is then resorbed by bone-resorbing cells (osteoclasts) that remove both calcified cartilage and mineralized bone. Finally, cells called osteoblasts build new bone to replace the resorbed calcified cartilage. This is one process that increases the size of the skeleton [10].

Because mineralized tissue is subject to damage and is metabolically active, mineralized bone is continually remodeled by the action of the basic multicellular unit (BMU) [11]. The BMU is composed of two cell types: bone resorbing osteoclasts and bone building osteoblasts. In the accepted model of bone resorption, the osteoclasts form a sealed resorption zone at the bone surface. The acidic environment of this sealed-off zone generates a “resorption pit” as it dissolves the bone mineral, subsequently releasing enzymes into the pit to digest the exposed collagen. Once the sealed, ruffled border of the resorption zone is broken, the osteoclasts can migrate to form a new resorption zone elsewhere. Curiously, the dissolved apatite mineral does not spontaneously re-precipitate within the resorption pit, even when the newly reopened zone returns to a neutral pH.

The void left by the excavating osteoclasts is filled with new bone formed by the osteoblasts. These bone-building cells lay down osteoid that in humans mineralizes 15 to 20 days later, a poorly understood period known as the mineralization lag time [12]. One unresolved question concerns the whereabouts of the apatite mineral components after they dissolve during bone resorption and before they reappear as new mineral within new bone. Bone tissue formation is understood to be a sequential process, but a clear understanding of the mechanism(s) in the early stages of mineralization has been elusive [13], [14].

This study revisits previously unexplained observations of early mineralization in fresh specimens of mineralizing bone and calcifying cartilage that have been processed with non-aqueous methods, as well as a previously published hypothesis linking bone mineralization to polyphosphate (polyP) ions. PolyPs and pyrophosphates (P_2_O_7_)^4−^ are known to be apatite crystal growth inhibitors [15]–[17]. Nevertheless, we propose that the production of polyPs, which form strong complexes with divalent cations such as calcium [18], can lead to a high local accumulation of total phosphate and calcium that exceeds the supersaturation limit of apatite without triggering the spontaneous precipitation of any apatite crystals. The controlled destruction of polyPs and subsequent production of Pi and free calcium can likewise exceed the local apatite supersaturation limit, only in this case it favors apatite mineral formation.

### Polyphosphates

Polyphosphates are synthesized by the condensation of phosphate species through dehydration processes such as heating [19], [20] or enzymatic condensation [21]. In this context, condensation means the merging of two orthophosphates, one orthophosphate and one polyP, or two polyP molecules to yield one longer polyP molecule and water. The condensed phosphate ions are linked together as polymers through phosphoanhydride (P-O-P) bonds. Unlike silicate ions, which condense in three-dimensional networks, linear polyP ions are the common species in aqueous environments [22].

Thermodynamics favor the hydrolytic degradation of P-O-P bonds exposed to water [20], resulting in the formation of two shorter polyP chains, or, more commonly, the formation of an orthophosphate ion and a shorter polyP chain. Interestingly, the kinetics of polyP hydrolytic degradation are slow at neutral pH and ambient temperatures [19]. This property suggests that the phosphoanhydride bond is an excellent candidate for enzymatic control. Researchers continue to identify enzymes responsible for the production (polyphosphate kinases) and degradation (polyphosphatases) of polyP through cleavage of orthophosphate (Pi) ions from the ends of polyP chains (exopolyphosphatases) or cleavage in the middle of polyP chains (endopolyphosphatases) [23]. PolyP complexes have been identified in eukaryotic membranes [24], mammalian cells and tissues [25], human platelets [26], prokaryotes, fungi, plants, and animals [23]. Many of the biochemical roles that polyPs play also have been studied [23], [27]. These roles range from energy production and cation sequestration to blood coagulation and fibrinolysis [28]. The participation of polyPs in bone mineralization has been previously proposed [29] but remains unexplained. PolyPs also have been identified in yeast mitochondria [30], where they serve as a bioavailable phosphate reserve—a role predicted decades ago [31]. Additionally, polyPs have been identified within dense “volutin” granules found in many microorganisms [32]; “electron-dense” granules that contain polyP have been recently linked to apatite biomineralization processes [33], [34].

### Electron-dense, amorphous (non crystalline) calcium phosphate granules

Electron-dense granules have been identified in a wide range of organisms [35] and were summarized in a recent review by Ryall [36]. Numerous types of calcium-containing, electron-dense granules exist in biology, some of which contain calcium, phosphate, magnesium, and carbonate. The list of proposed functions for these granules includes waste disposal, osmoregulation, excretion of excess ions, calcium/phosphate/carbonate storage and mobilization, and skeletal function [36]. In the 1960s, scientists studied amorphous and electron-dense granules containing calcium and phosphate and appearing within mitochondria [37], [38]. The participation of mitochondria in apatite biomineralization has been proposed previously, but never explained [38]–[40].

### Mitochondria, polyphosphates and biomineralization

PolyPs are known to be employed by mitochondria as a means for storing relatively high concentrations of bioavailable orthophosphate without precipitating apatite crystals [30]. Electron-dense granules that ranged from 500–1000 Å within rat liver mitochondria were determined to contain calcium and phosphate at concentrations of ∼0.5 M, exceeding the solubility product of calcium phosphate and calcium pyrophosphate minerals [41], [42] at neutral pH [37]. In 1964, Lehninger's group used X-rays to show that these calcium- and phosphate-containing granules were non-crystalline (amorphous) [38].

Localized and unstable granules containing high concentrations of calcium and phosphate were also identified in the mitochondria of proliferating and hypertrophic mineralizing chondrocytes [43], [44]. Their location suggests that these granules are associated with the calcification process. This observation required frozen, fresh, or non-aqueous sample preparation, however, because unknown chemical reactions induced by aqueous methods interfered with the granule detection [43].

### Skeletal tissue and electron-dense granules are affected by sample preparation

The study of tissue mineralization has been hindered by sample preparation techniques because skeletal minerals are labile and often affected by aqueous processing methods [45]–[47]. Previous electron microscope examination of unstained growing bone processed with *non*-aqueous methods has revealed electron-dense “granules” within the mineralizing osteoid matrix, including within the mitochondria of osteoblasts, osteocytes and osteoclasts [47]–[49]. Similar granules with a high total calcium and phosphate content, an unexpected amorphous structure, and a low calcium to phosphorus ratio were previously detected by electron microscopy in rat growth plate cartilage [43]. The study authors, however, did not explain either the amorphous nature of these granules or their instability when exposed to aqueous processing methods [43]. Mitochondrial electron-dense granules that proved sensitive to calcium fixation were identified in chondrocytes; adding calcium to the osmium-fixation processing method enhanced the granules' stability [44].

In the late 1960s, researchers reported a transient histological staining of the growth plate that was dependent on the sample preparation method. Studies demonstrated transient staining of fresh rat epiphyseal cartilage in the matrices of resting and hypertrophic zones, as well as in proliferating and hypertrophic chondrocytes, while no such staining was observed with cartilage that had been frozen and thawed or processed with standard histological methods [50], [51]. It was proposed that a change in the unstable granules might factor into the calcification of cartilage. Electron microscopy of the epiphyseal growth plate suggested that the mitochondrial granules composed of unstable, amorphous calcium salts were likely candidates for the nucleation sites necessary for skeletal mineralization [44].

### Skeletal mineralization and alkaline phosphatase

One of the putative organic components of the skeletal mineralization process was first identified in 1923. Robison [52] suggested that a phosphatase enzyme located within calcifying cartilage could yield free phosphate ions, implying that phosphatase is “omnipresent and essential in calcifying areas” [53]. Tissue-nonspecific alkaline phosphatase (TNAP) is an isoenzyme of alkaline phosphatase [54], which is expressed in liver, kidney, and skeletal tissues; specific examples include hypertrophic chondrocytes [55] and osteoblast cells [56]. TNAP is also presumed to be involved in skeletal mineralization [57], but its substrate(s) is not well defined. Bone and cartilage mineral formation is associated with the presence of TNAP, which was proposed to control the concentration of orthophosphate available for apatite mineral formation [58]. An enzymatic characterization of alkaline phosphatase from bovine epiphyseal cartilage noted the hydrolytic degradation of a variety of phosphate esters, as well as of ATP and pyrophosphate [59].

### Summary

In 1961, Fleisch and Neuman proposed that the role of phosphatase in bone is to destroy a mineralization inhibitor, “perhaps a polyphosphate” [60]. Herein, we present experimental data that support their hypothesis, and expand their proposal to include the hypothesis that polyP formation provides a mechanism for accumulating phosphate, thus controlling apatite supersaturation at locations such as sites of cartilage calcification and bone resorption. We also demonstrate that polyPs are located in areas of resorbing bone and calcifying cartilage, and that TNAP cleaves orthophosphate from polyphosphate. Furthermore, we show that exogenously applied, intestinal alkaline phosphatase (IAP), an enzyme known to cleave orthophosphates from polyphosphates [61], decreases the polyP content within the growth plate of murine vertebral body sections *in situ*. Finally, we demonstrate that polyPs reduce hydroxyapatite supersaturation by sequestering free calcium and also adsorb to hydroxyapatite surfaces *in vitro*.

Our hypothesis is that apatite is the mineral component of bone because enzymatic action can control apatite supersaturation at neutral pH by directly controlling orthophosphate ion activity. Vertebrate mineralization may then be modulated through the synthesis and hydrolytic degradation of polyphosphate ions.

## Results

### Electron-dense, calcium- and phosphate-rich granules are present at bone resorption sites

**Figure 1:**
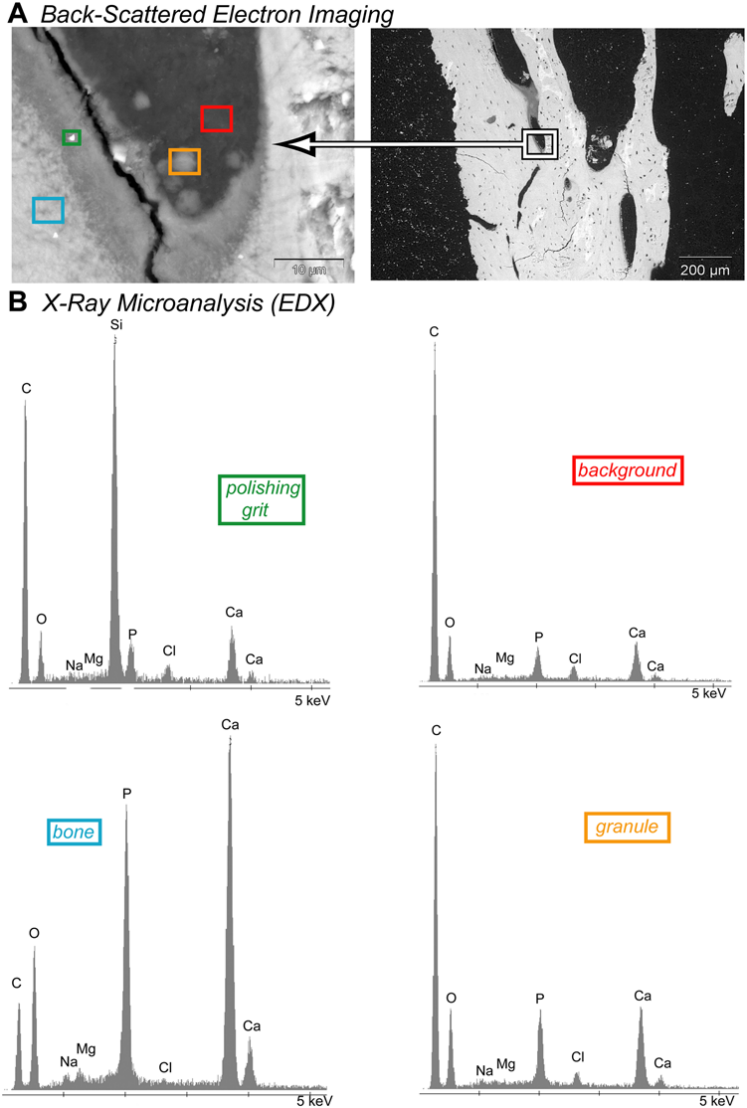
Electron-dense granules identified in resorbing bone contain P and Ca. (A) Back scattered electron (BSE) images (high and low magnification, left and right respectively) and (B) energy dispersive x-ray analysis (EDX) of acetone-dehydrated, Spurr®-embedded, 9-month-old guinea pig tibial cortical bone, showing intensity (y-axis) vs. emission energy (x-axis). Each spectrum corresponds to analysis of the colored square region of interest (ROI) defined in (A). Red ROI is background (low Ca, P), blue ROI is mineralized bone (high Ca, P), yellow ROI is an electron-dense granule (intermediate Ca, P), and green ROI is a polishing grit artifact (Si).

Using back-scattered electron (BSE) imaging, we observed electron-dense granules (<5 µm in diameter) within a location identified as a resorption pit based on its tunneled morphology in the cortical shell of an undecalcified, 9-month-old guinea pig tibia (Figure 1A). Energy dispersive X-ray (EDX) analysis of the granules found in this resorption zone indicated the presence of calcium, phosphorous, and oxygen atoms. A granule is indicated within the orange square in Figure 1B. The Pi and calcium signal intensities of the granules were intermediate to the background (red square) and the mineralized bone (blue square).

### Identification of polyphosphates in a basic multicellular unit by fluorescence microscopy

**Figure 2:**
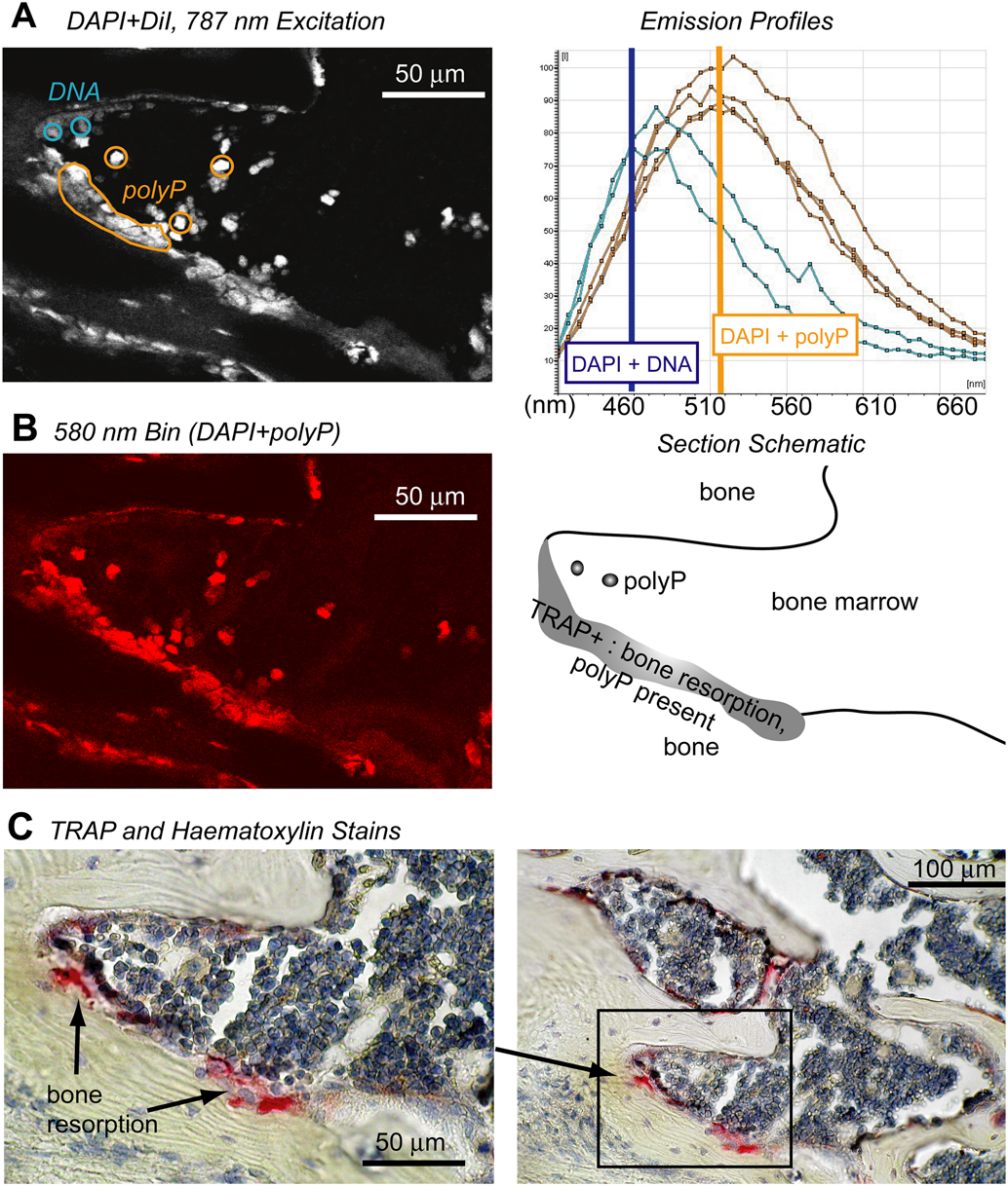
Detection of polyP in a bone resorption site of an EDTA-decalcified, 3-month-old murine vertebra. (A, left) Confocal fluorescence image (400–700 nm) from a 5–10 micron bone section stained with DAPI and exposed to multiphoton excitation (787 nm). (A, right) Spectral scans of imaged region (A, left) were acquired in 20 nm bins. Emission intensity was plotted for each of the indicated ROI. Blue ROI: DAPI-DNA emission. Yellow ROI: DAPI-polyP emission. (B, left) The 580 nm bin emission for the same image captured in (A) spatially resolves DAPI-polyP distribution. (B, right) Schematic identifies relevant fluorescent regions (A, left) within the resorption zone. (C) The same bone section was subsequently stained for TRAP and counterstained with haematoxylin (an aqueous process, thought to accelerate hydrolytic degradation of polyP) to confirm the presence of osteoclasts (red staining) at the resorption site (left and right images correspond to high and low magnification, respectively).

Figure 2A shows an example of the spectrum-wide emission from DAPI-stained vertebral bodies using laser-scanning confocal microscopy. Fluorescence was collected between 400–700 nm; after scanning across the same spectral range in 20 nm bins, we selected various regions of interest and plotted the emission spectrum (Figure 2A, right). DAPI-polyP complexes were identified within granules located in regions of bone resorption by their specific emission wavelength at 520 nm (yellow) [62], [63], whereas regions lacking polyP featured a prominent peak emission near 460 nm—a characteristic emission wavelength for DAPI-DNA (blue) (Figure 2A, vertical lines). The image in Figure 2B (left) was recorded at the 580 nm emission bin, and spatially shows the regions with a DAPI-polyP complex emission. Regions that fluoresced at 580 nm included granules set back from a resorption pit, as well as regions associated with bone resorption (see diagram in Figure 2B, right). Regions of bone resorption were confirmed by subsequent staining of the same section for tartrate-resistant acid phosphatase (TRAP, red), which is a marker for osteoclasts [64], and counterstaining with haematoxylin (Figure 2C).

### Detection of polyphosphates in calcifying cartilage by fluorescence microscopy

**Figure 3:**
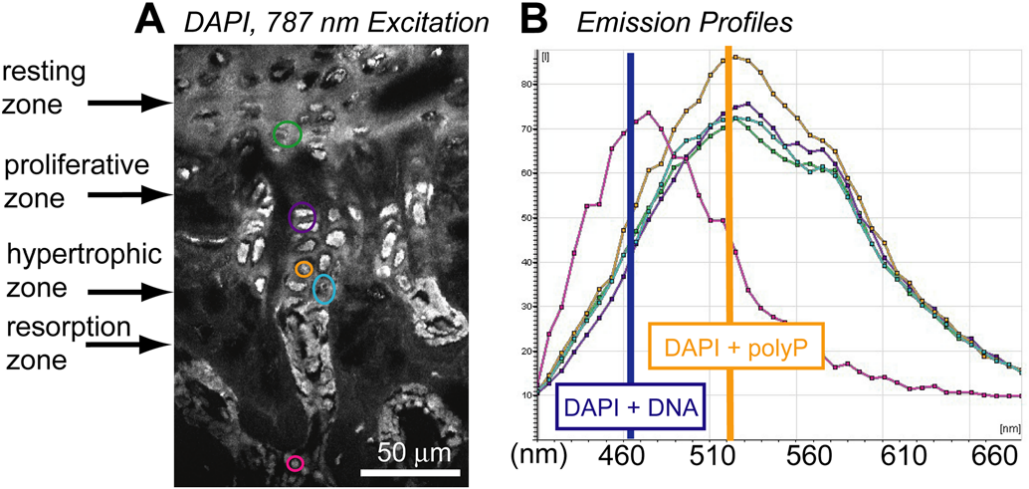
DAPI identified polyP in vertebral growth plates. Growth plate sections (EDTA-decalcified, 3-month-old murine vertebral body) cut under non-aqueous conditions and treated with DAPI. (A) Fluorescence emission (400–700 nm) imaged upon multiphoton excitation with emission wavelength scan analysis (B) by color-defined ROI. Red ROI in sub-chondral bone: emission wavelength profile corresponding to that of DAPI bound to DNA, not polyP. Other ROI's: emission wavelength profile with a maximum at ∼520 nm, corresponding to that of DAPI bound to polyP.

We also detected polyP in 3-month-old murine vertebral body growth plates that had been decalcified in cold EDTA, dry-sectioned, stained with DAPI (50 µg/mL), and analyzed by fluorescence microscopy (Figure 3A). PolyP was detected within the resting zone (green circle), hypertrophic matrices (blue circle), proliferating chondrocytes (purple circle), and hypertrophic chondrocytes (orange circle) by the presence of DAPI-polyP fluorescence peaks at 520 nm (Figure 3B).

### Detection of transient toluidine-blue stained regions in the growth plate

**Figure 4:**
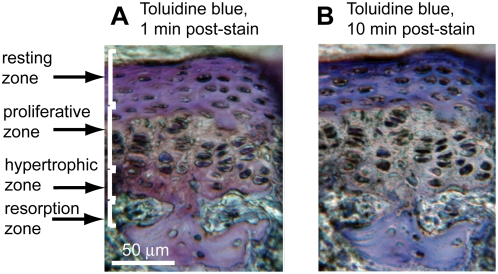
Transient staining of vertebral growth plates with toluidine blue. Growth plate sections (EDTA-decalcified, 3-month-old murine vertebral body) (A) 1 minute and (B) 10 minutes after toluidine blue staining. Note reduced staining of hypertrophic zone matrix after 10 minutes.

For this study, we repeated the transient staining of fresh epiphyseal cartilage reported by Hirschman [51]. Figure 4A shows the toluidine blue stain one minute after staining, while Figure 4B shows fading of the stain in the hypertrophic zone matrix of the same section 10 minutes after staining. The resting zone stain also transformed from purple to blue. The growth plate regions that were transiently stained with toluidine blue are similar to the regions that fluoresced with the DAPI-polyP signature (∼520–580 nm emission maximum, Figure 3).

### Reduction of polyphosphate content in the growth plate by intestinal alkaline phosphatase

If the 520–580 nm DAPI-polyP signal in vertebral growth plates partially identifies polyPs, then application of intestinal ALP (to hydrolyze polyPs) should alter the emission profile. The DAPI emission spectra of ALP-treated and non-ALP-treated control sections were retrieved from subregions of interest that generally encompassed the growth plate.

**Figure 5:**
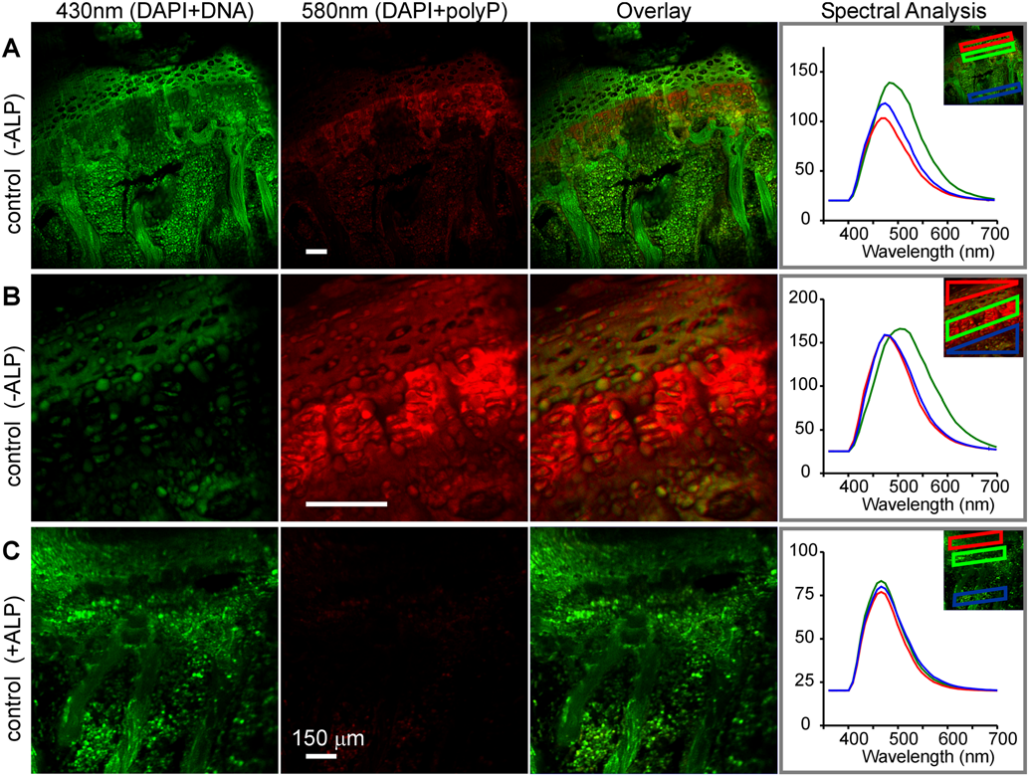
Resolving polyP distribution by spectral isolation of DAPI 580 nm emission and attenuation by alkaline phosphatase (ALP) treatment. (A) Control growth plate section; (B) a different control growth plate section (higher magnification); (C) ALP-treated (+ALP) growth plate section. First column, green: 430 nm emission (DAPI-DNA) Second column, red: 580 nm emission (DAPI-polyP). The third column is an overlay of the 430 and 580 nm images to show the spatial distribution of polyP that occurred primarily in the hypertrophic zone of control sections but was absent in ALP-treated sections. The spectral analyses in the last column show profiles for the various ROI outlined in the smaller inset image. Growth plate regions analyzed included the region above the hypertrophic matrix (red ROI), in the hypertrophic matrix (green ROI), and in the bone (blue ROI). The emission profile of each control (−ALP) hypertrophic matrix (green ROI) shows a shift to a higher wavelength emission compared to the other −ALP regions, suggesting the presence of polyP. All regions analyzed in +ALP sections showed an emission profile similar to that of DAPI-DNA, suggesting polyP was hydrolyzed from the hypertrophic matrix after exposure to active ALP. All scale bars represent 150 µm.

**Figure 6:**
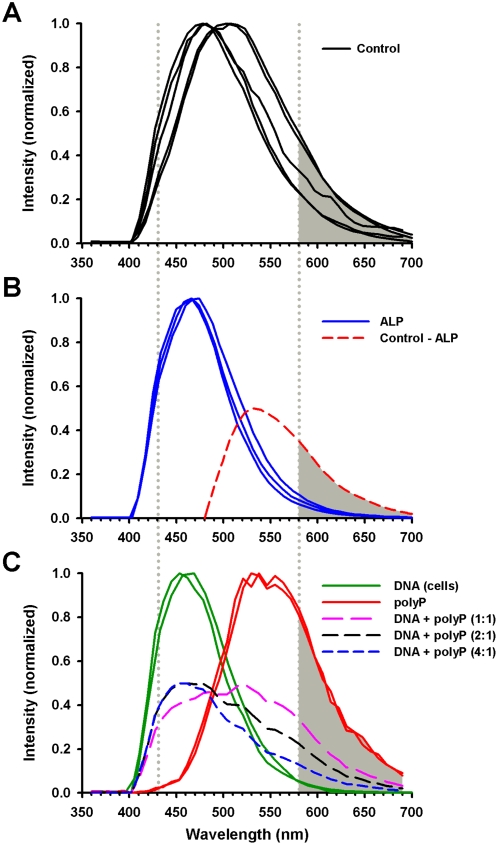
DAPI emission in the hypertrophic zone is a convolution of DAPI-DNA and DAPI-polyP emissions. (A) −ALP (control) emission spectra from control sections show a large fluorescence contribution above 580 nm. (B) Sharper and lower wavelength emission profile in sections treated with ALP to hydrolyze polyP. The spectral shift due to ALP treatment (control – ALP) is indicated by the red dashed line. (C) Emission profile of polyP-poor murine brain cells (green lines) contrasts with that of rightward-shifted emission from synthetic polyP (red lines). Summing DAPI-DNA-rich and polyP curves (1∶1 ratio, peak re-scaled to 0.5), yields a complex, wide emission profile (dashed pink line); as the DNA∶polyP contribution is increased (2∶1 and 4∶1), the composite emission profile shifts to the left and the peak width narrows.

Figure 5 compares representative individual scans and overlays of emissions at a wavelength indicative of DAPI-DNA (430 nm, green) and DAPI-polyP (580 nm, red) for the control (−ALP; Figure 5A,B) and ALP-treated (+ALP; Figure 5C) murine vertebral body sections. When we displayed the DAPI fluorescence images collected at 580 nm with respect to 430 nm, we found a unique spatial distribution for the two wavelength ranges (Figure 5, overlay column). Thus, the DAPI emission above 580 nm may be a good index of polyP distribution. The spectral analyses on the right show representative profiles for the growth plate regions above the hypertrophic matrix (resting zone, red outline in inset), within the hypertrophic matrix (green outline), and within the bone (blue outline). The emission profiles of the hypertrophic matrix in control sections (Figure 5A,B) showed a shift to longer wavelengths compared to the other regions, suggesting the presence of polyPs. An overlay of all control profiles over the hypertrophic matrix region (Figure 6A) features a long tail of appreciable DAPI fluorescence extending beyond 580 nm.

Compared to control sections, those sections treated with ALP (including the one shown in Figure 5C) had limited DAPI emission above 580 nm in all regions analyzed. The spectral curves for the hypertrophic matrix region from all ALP-treated sections are overlaid in Figure 6B. We observed virtually no emission above 580 nm after ALP treatment, suggesting that polyP was not detectable in the hypertrophic matrix after exposure to active ALP. Additional analyses suggest that the ALP treatment reduced the spectral emission to a simpler profile that is more similar to that of DAPI-DNA. Table 1 summarizes the effect of exposing sections of murine vertebral growth plate (hypertrophic matrix region) to ALP (n = 3) or buffer (control: n = 5, including one section treated under ALP-inactivating, room temperature conditions) on the DAPI fluorescence emission spectra. ALP application significantly reduced the intensity of the DAPI emission spectra at 520 nm with respect to the emission at 430 nm. The position of maximum intensity of the ALP-exposed sections (Figure 6B) was shifted closer to the emission of DAPI-DNA (460–465 nm). The FWHM of the emission spectra was decreased by ALP exposure, indicating a reduction in intensity of one of the convoluted DAPI-DNA or DAPI-polyP curves. In summary, ALP-treated sections showed a combination of effects: a reduced DAPI-polyP∶DAPI-DNA ratio (520∶430 nm), a peak position shift to lower wavelengths, and a reduction in the FWHM, consistent with the reduction in DAPI-polyP emission.

**Table 1:** Effect of ALP exposure to DAPI emission spectra of the murine growth plate.

Test	520/430 nm intensity ratio	Position of maximum intensity (nm)	FWHM (nm)
−ALP (control)	2.14±0.84	490±14	120±10
+ALP	0.64±0.21	469±5	88±4
p (t-test)	0.02	0.05	0.01

In the control hypertrophic matrix, the shift to a higher and wider wavelength profile for DAPI fluorescence appears to represent a composite of DNA and polyP spectral emissions. For instance, mathematically subtracting an ALP-treated spectral curve from a control spectral curve yielded an emission curve with a peak near 540 nm (Figure 6B, dotted line). We performed two additional experiments to further characterize the spectral signatures of DAPI bound to DNA instead of to polyPs. First, we acquired the emission spectra for DAPI-DNA using murine brain cells as a DAPI-DNA baseline that did not contain appreciable levels of polyPs. Next, we applied DAPI to synthetic polyPs in solution, mounted the mixture on a slide, and collected the emission spectrum (Figure 6C). The DAPI-polyP curve (red) was rightward shifted compared to DAPI-DNA (green) and also matched closely with the subtracted curve obtained from the vertebral growth plates (control minus ALP, Figure 6B dashed line). Interestingly, adding the DAPI-DNA emission curve to the synthetic polyP curve at a 1∶1 weighting yielded a very wide curve (pink dashed line, scaled to 0.5). As the DNA∶polyP contribution increased (2∶1 black dashes and 4∶1 blue dashes), however, the resulting spectral curve narrowed at the peak and shifted to lower wavelengths; this effect appears analogous to what we observed in the ALP experiment (Figure 6B, blue). Once again, the 580 nm intensity proved to be a good index of polyP contribution (vertical line in Figure 6C). The more variable emission profiles in the hypertrophic matrix region between sections may represent a submixture of polyP and DNA staining and/or difficulty in maintaining intact polyPs during sample preparation. Regardless, the simplest interpretation of the bone tissue data is that ALP treatment reduced the emission of the higher wavelength component that corresponds to polyPs.

### Tissue-nonspecific alkaline phosphatase hydrolyzes polyphosphates, producing orthophosphate ions

**Figure 7:**
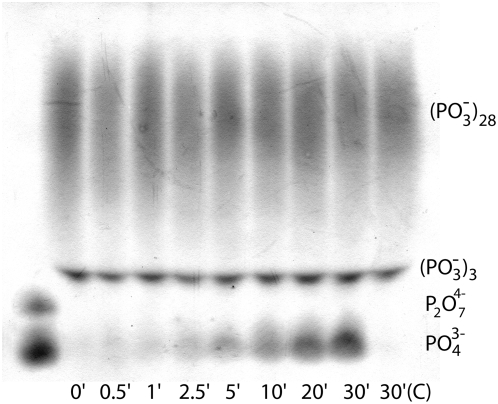
TNAP cleaves Pi from polyP. Separation of synthetic polyP exposed to TNAP by polyacrylamide gel electrophoresis. TNAP exposure times were 0.5, 1, 2.5, 5, 10, 15, 20, and 30 minutes. Right lane is TNAP- (control) for 30 minutes (C) and the far left lane is a pyrophosphate (P_2_O_7_)^4−^/Pi ladder. Synthetic polyP contains a range of polyP species, from (PO_3_^−^)_3_ (3 condensed Pi units) to polyP chains longer than 28 Pi units.

Figure 7 shows the result of an *in vitro* assay of bovine kidney ALP, a TNAP isoenzyme, with long chain polyPs. Electrophoresis of the enzymatic digestion products demonstrated that this enzyme cleaves Pi from synthetic polyPs (average chain length of 25 Pi units, with some polyP species as short as 3 Pi units). The Pi concentration increased with time in the +ALP experiment, while no notable Pi was detected in the −ALP (control) experiment, even at the longest time point (30 minutes). Densitometry measurements of the bands showed an increase in Pi and decrease in polyP concentrations over time (Supplementary Information, Figure S1).

### Polyphosphates adsorb to hydroxyapatite

The adsorption experiment showed that polyPs adsorb to HAP (see Supplementary Information Figure S2).

### Polyphosphate addition reduces hydroxyapatite saturation

To calculate the effect of Pi and polyP addition on the HAP relative saturation, we measured the free calcium, Pi, and pH, and used those values to calculate the apparent HAP saturation.

**Figure 8:**
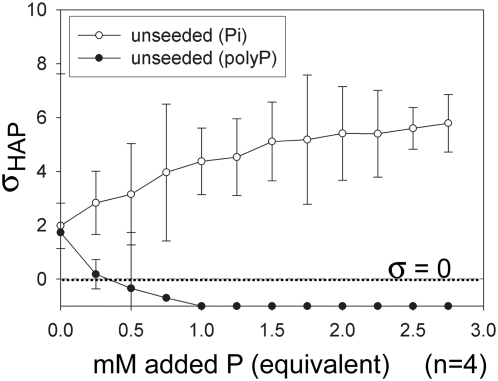
The effect of adding P to a HAP-saturated, unseeded solution depends on the speciation of P. Increasing the equivalent P concentration by adding Pi to a solution saturated with HAP and without any HAP crystals in solution (unseeded) increases the relative HAP saturation (σ_HAP_>0, open circles). Addition of P as polyP (unseeded) decreases the apparent saturation of HAP to an undersaturated state (σ_HAP_<0, closed circles). PolyP addition sequesters calcium, reducing calcium activity in solution without changing the free Pi concentration appreciably. Although supersaturated with respect to apatite when calculated with the total calcium and phosphate concentrations, HAP solids would be expected to dissolve when calcium sequestered by polyphosphates effectively generates a system undersaturated with respect to HAP.

Figure 8 shows the effect of increasing Pi concentration (open circles) or polyP concentration (filled circles) on the apparent relative saturation of HAP (σ_HAP_) in the unseeded experiments. σ_HAP_ increased with increasing Pi concentration. Raising the equivalent P concentration by adding polyP lowered σ_HAP_ below the HAP saturation level (σ_HAP_<0) because polyP chelates calcium.

## Discussion

### Electron-dense, calcium- and phosphate-rich granules are present at sites of bone resorption

Calcium- and phosphate-containing granules observed within resorption pits in undecalcified bone sections and possessing an intermediate electron density (between that of bone and unmineralized tissue) may be related to similar calcium- and phosphate-rich granules previously detected in mineralizing bone processed under non-aqueous conditions and likewise possessing an intermediate electron density (between that of unmineralized collagen and apatite) [48]. Unstained, electron-dense granules in 6-day-old mouse rib have been observed in bone sections prepared under non-aqueous conditions [48]. The granules observed in this study (in the range of a few thousand Å) are larger than previously reported mitochondrial electron-dense clusters in mineralizing osteoid (ranging from 400–1000 Å in diameter) that are composed of 50–100 Å electron-dense particles in young rats. These “mitochondrial granules” were detected in osteocytes (bone cells), osteoblasts, extracellular matrices, and osteoclasts [48].

Mitochondria are known to harbor high concentrations of bioavailable Pi by storing them as polyP [30], [65], [66]. A large number of mitochondria have been observed in osteoclasts [67], [68]. Although the resolution of our technique does not permit detection of polyP within the mitochondria of bone-resorbing osteoclasts, the ability of mitochondria to store Pi as bioavailable polyP suggests that these organelles may be employed by osteoclasts to scavenge and store the Pi released by apatite dissolution during bone resorption.

Landis used electron microscopy to measure the size of electron-dense granules and calculate the Ca∶P molar ratio in granules located within mitochondria (Ca∶P = 0.8–1.1), osteoid (Ca∶P = 1.2–1.3), and mineralized bone (Ca∶P = 1.4–1.5) [47]. He noted that the lower Ca∶P ratio of the electron-dense granules was similar to that measured for monetite (CaHPO_4_; molar Ca∶P = 1.0) and calcium polyP (Ca(PO_3_)_2_)_n_; molar Ca∶P = 0.5). A Ca∶P molar ratio of 0.5 is also expected for a calcium polyP complex (Ca(PO_3_)_2_)_n_.

Producing polyP from Pi is an effective way to decrease the free (available for chemical reaction) Pi concentration and calcium ion activity. Osteoclastic production of polyP from Pi retrieved from dissolved apatite increases total phosphate while avoiding a rise in calcium phosphate mineral saturation. PolyP formation from the dissolved apatite within the acidic resorption zone decreases the saturation of calcium phosphate minerals such as brushite (CaHPO_4_·2H_2_O) that would otherwise be expected to remain stable in the acidic resorption zone. Granules containing polyP, which could also contain sequestered calcium, are capable of co-localizing high concentrations of the major ionic components of apatite without precipitating apatite crystals within volumes of neutral pH either inside or outside the osteoclast.

**Figure 9:**
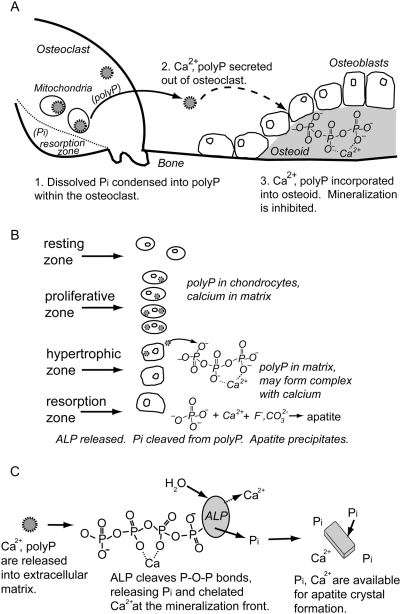
Schematics of hypothesized roles of polyP in the mechanisms of phosphate transport in remodeling bone and calcifying cartilage, and apatite crystal precipitation. (A) Hypothesized mechanism of phosphate metabolism and transport as polyP in osteoclastic bone resorption and bone mineralization. Apatite mineral dissolution in the osteoclast resorption zone increases the concentrations of free Pi and Ca^2+^. The mitochondria may scavenge the Pi and condense them into polyP that may also sequester Ca^2+^. Amorphous granules containing total concentrations of Ca^2+^ and Pi higher than the saturation of apatite are formed and may be transported out of the osteoclast. The granules may be transported to or produced within the osteoblasts that build new bone. The osteoblasts may embed the granules in osteoid (new, unmineralized bone). The reaction of these granules with alkaline phosphatase (ALP, present at the membrane of osteoblasts) cleaves Pi from polyP and would increase the free Pi concentration and release any sequestered Ca^2+^. The increase in free Ca^2+^and Pi could exceed the saturation for apatite and result in apatite mineral formation. (B) Hypothesized role of polyP in growth plate mineralization. PolyPs are condensed within the proliferative zone chondrocytes, possibly by mitochondria. PolyP-containing, amorphous, electron-dense granules are transported to the chondrocytes in the hypertrophic zone matrix. PolyP ions may chelate with Ca^2+^, resulting in high total concentrations of Ca^2+^ and Pi locally but no apatite formation. ALP cleaves Pi from the polyPs, increasing the free Pi concentration and releasing any sequestered Ca^2+^. The increase in free Pi and Ca^2+^concentrations may exceed the saturation of apatite, resulting in apatite mineral formation. (C) Hypothesized process of precipitating apatite crystals from calcium polyP complexes with ALP. Calcium polyP complexes release sequestered Ca^2+^ and Pi when ALP cleaves Pi from the end of polyP chains. The increased free concentrations of Ca^2+^ and Pi raise the apatite saturation, favoring apatite precipitation.

Previous research showed that the calcium and phosphate content of electron-dense, non-crystalline granules identified in mitochondria exceeded the solubility product of calcium phosphate [37] without forming crystalline apatite mineral. One possible explanation for these amorphous granules being supersaturated with respect to apatite is that the measured Pi was originally present as polyP. Therefore, if the phosphate is present as polyP, the formation of crystalline apatite may not be thermodynamically favored. We hypothesize that polyPs produced by the mitochondria in osteoclasts permit the storage of high concentrations of bioavailable Pi without forming apatite, and allow for the transport of high concentrations of phosphate to locations such as osteoid (Figure 9A).

Polyphosphates and pyrophosphates are known to inhibit the growth of apatite mineral [15]–[17]. The destruction of pyrophosphate by TNAP in the presence of Type I collagen has been shown to induce mineralization [69]. While polyP may likewise act as an apatite growth inhibitor, polyP-calcium complexes can also form concentrated amorphous granules with a composition of calcium and phosphate (0.5 M) that is greater than the solubility product of apatite [37] or calcium pyrophosphate [41], [42]. This ability of polyP to amass high concentrations of calcium and phosphate permits the accumulation of the major components of apatite for its controlled mineralization by ALP in neutral pH environments.

### Identification of polyphosphates in calcifying cartilage by fluorescence microscopy

Shapiro and Boyde [70] proposed that the rate-limiting step for cartilage calcification was the transport of Pi from the cartilage cells to the calcifying cartilage matrix. Electron-dense “spherules” ranging in size from 200–800 Å were also identified within mitochondria of the chondrocytes (cartilage cells) in the proliferative and hypertrophic zones of fresh, stained, tibial epiphyseal plates and occipital bones of mice and rats. Likewise, such spherules were spotted in the extracellular regions of the proliferative and hypertrophic zones [43]. These granules were not observed when the tissue was prepared with aqueous methods, suggesting they may have been composed of polyP. Labile calcium and phosphate-containing spherules were also detected within the cytoplasm of chondrocytes and adjacent to hypertrophied chodrocytes in the matrix of fresh, calcifying cartilage [71]. With the detection of polyP in these specific regions of the growth plate, we hypothesize that polyP granules produced by the proliferating chondrocytes are secreted into the calcium-rich lower hypertrophic zone matrix (Figure 9B). Accelerated hydrolytic degradation of polyP with ALP in polyP-calcium complexes would provide a source of Pi and calcium for apatite precipitation (Figure 9C).

Landis and Glimcher previously quantified the calcium and phosphate ratio for different calcium phosphate minerals, bone mineral, and electron-dense granules detected in calcifying cartilage [43]. They demonstrated that these dense granules detected in the proliferating and hypertrophic chondrocytes of the growth plate exhibited no crystalline structure and contained calcium and phosphorus in molar ratios ranging from 0.80±0.05 to 1.07±0.24. Within the extracellular matrix, the Ca∶P molar ratio increased from 0.88±0.12 in the mid proliferating zone to 1.51±0.09 in the zone of calcifying cartilage. The Ca∶P molar ratio in the unmineralized areas of the growth plate fell between the values measured for linear polyphosphate ((Ca(PO_3_)_2_)_n_; 0.50) and brushite (CaHPO_4_; 1.03). The Ca∶P ratio in calcifying cartilage is closer to that of hydroxyapatite (1.62). Another study of the epiphyseal growth plate showed that Pi-containing matrix granules were distinct from Ca-rich sites in the unmineralized upper regions, and calculated Ca∶P ratios of 1.0 near the zone of provisional calcification [72].

The measurement of Ca∶P molar ratios of less than 1 in electron-dense, amorphous granules in the proliferating extracellular zone and in the proliferating and hypertrophic chondrocytes can be explained by the presence of a polyP component, because polyP can form calcium complexes with Ca∶P ratios as low as 0.5.

### Toluidine blue staining of the growth plate

We successfully reproduced the transient toluidine blue staining reported by Hirschman and McCabe to compare it with DAPI staining of polyP in the growth plate. Toluidine blue undergoes a metachromatic shift when complexed with polyP; the size of the shift depends upon the concentration of polyanionic charges [73], [74]. Therefore, the different metachromatic shifts of toluidine blue in the fresh rat epiphyseal cartilage may have been due to polyP ions of different chain length and/or concentrations. Fading of the toluidine blue stain in the matrices of the resting and hypertrophic zones may have been caused by a loss of the polyP substrate. This loss of substrate would have resulted from polyP hydrolytic degradation when the thin tissue sections were exposed to water, because polyPs are thermodynamically favored to undergo hydrolytic degradation [19].

Although toluidine blue stain is not specific for polyP, previous research found that the areas of the fresh rat epiphyseal cartilage that stained with toluidine blue also stained with neutral red and methylene blue [50], both of which also stain polyP [23]. These observations further support the finding of polyP in these zones with DAPI and fluorescence microscopy.

### Reduction of polyphosphate content in growth plate sections with intestinal alkaline phosphatase

The application of intestinal ALP to an EDTA-demineralized, dry-cut section of murine growth plate reduced the intensity of the DAPI-polyP emission spectra. We conclude that the intestinal ALP increased the hydrolytic degradation rate of polyP present in the section. The reduction in polyP content resulted in a decreased intensity of the DAPI-polyP emission spectrum near 520 nm, sharpening the total emission spectrum towards a DAPI-DNA emission curve. Given the low resolution of this technique, we used the emission from a region of interest spanning the zones of the growth plate to measure the effect of ALP addition on the emission curve.

We determined that the critical wavelength bin between 580–700 nm is a reliable and specific measure of DAPI-polyP distribution. DAPI fluorescence from both the ALP-treated sections and also the polyP-poor signal measured in murine brain cells showed a negligible contribution above 580 nm. We were able to image the 580 nm DAPI-polyP emission and found the strongest fluorescence within the hypertrophic matrix region of the vertebral growth plate (Figure 5). It should be possible to use this approach to further study the process of vertebrate skeletal mineralization.

### Tissue-nonspecific alkaline phosphatase hydrolyzes polyphosphates, producing orthophosphate ions

Figure 7 indicates that Pi is a hydrolytic degradation product of polyP by TNAP. The action of bovine TNAP on inorganic polyP, cleaving Pi from the ends of polyP chains, classifies this enzyme as an exophosphatase [23].The production of only Pi from Ca-polyP granules by TNAP would increase the Pi concentration and calcium ion activity through the release of calcium ions from their chelation with polyP. In addition, the hydrolytic degradation of polyP present in calcium-rich environments, such as the growth plate, may also increase the Pi concentration, thereby raising apatite saturation and providing sufficient thermodynamic driving force for apatite crystal nucleation and growth (Figure 9C). Co-localization of polyP and ALP in the mineralization sites of remodeling bone and calcifying cartilage would suggest that apatite crystal nucleation may be a consequence of ALP controlling the hydrolytic degradation of polyP into Pi ions in the presence of free calcium.

### Polyphosphates can reduce hydroxyapatite (HAP) saturation

Increasing the free Pi concentration in a HAP-saturated, unseeded system increased the σ_HAP_. This is an expected result because free Pi concentration is proportional to σ_HAP_. The polyP adsorption experiment showed decreasing apparent σ_HAP_ in an HAP-saturated system that contained no HAP crystals (unseeded). The reduction in σ_HAP_ observed with increasing polyP concentration may be attributed to the formation of calcium-polyP complexes in solution. The ability of polyP to sequester calcium may reduce the activity (free concentration) of calcium ions in the initially HAP-saturated solution. The measurement of very low free calcium with increasing polyP addition (Figure S2) suggests that the additional polyP ions formed stronger complexes with calcium than with the colorimetric agent used to measure calcium concentration. The resulting reduction in calcium ion activity (α_Ca2+_) would decrease the HAP ionic product (IAP_HAP_→0), and hence reduce σ_HAP_ to values<0. In this induced undersaturated state, the HAP seed is predicted to dissolve.

During the seeded polyP adsorption experiment, HAP seed crystals dissolved as total calcium ion and hydroxyl concentrations increased with the increasing addition of polyP (Figure S2). Adding polyP to the HAP-saturated solution reduced the free calcium concentration, causing an undersaturation of the solution with respect to HAP and thus favouring the dissolution of the HAP seed.

Adding only polyP to a solution saturated with HAP is not expected to change the free Pi concentration appreciably. In an aqueous solution, however, thermodynamics predicts the spontaneous, hydrolytic degradation of polyP into Pi ions [19], although the kinetics are slow. Some polyP hydrolytic degradation in our experimental conditions may explain the increase in Pi concentration in the unseeded experiment (Figure S2), an observation that also would be explained by dissolution of HAP seed crystals.

These results suggest that a system containing polyP and sequestered calcium could exhibit low Pi and free calcium concentrations, indicating an undersaturated system with respect to HAP. PolyP hydrolytic degradation of the same system, however, would produce free Pi and calcium, revealing a system oversaturated with respect to HAP. This dichotomy may explain why previous researchers have observed amorphous, electron-dense granules with total calcium and phosphate concentrations exceeding the saturation of apatite, but never detected the presence of apatite crystals.

### Hypothesis

Polyphosphate ions identified at bone-resorbing osteoclastic sites, in the proliferating and hypertrophic zone chondrocytes, in the hypertrophic zone matrix, and postulated to be present in unmineralized osteoid may provide a phosphate reserve and mechanism for controlling biological apatite mineral deposition. We propose that polyphosphates are formed or incorporated within the skeleton where high local concentrations of orthophosphate exist and where mineralization is not desired. High concentrations of calcium and phosphate can be transported from bone resorption zones as bioavailable calcium-polyphosphate complexes while remaining below the saturation of apatite. If transported as polyphosphate ions, phosphate ions may be ferried to regions of high calcium concentration in pre-calcifying cartilage without risking apatite formation. When mineralization is desired, alkaline phosphatase would accelerate the hydrolytic degradation of polyphosphates, releasing orthophosphates and any sequestered calcium. At a neutral pH, this polyphosphate hydrolytic degradation reaction would increase the orthophosphate and free calcium concentrations, raising apatite saturation and supplying the driving force for biological apatite formation.

This flexible, enzymatic control of mineralization explains why the vertebrate skeleton is composed of apatite. Carbonates do not condense and silicates condense but are not hydrolytically degraded in biological systems. Phosphates are condensed by polyphosphate kinases and orthophosphates are produced by the hydrolysis of polyphosphates with alkaline phosphatase. The enzymatic control of orthophosphate activity provides a simple and efficient method for controlling apatite mineral formation in vertebrates. We therefore propose that polyphosphates are an efficient modulator of apatite biomineralization *in vivo*. Apatite mineralization and demineralization in growing, continually remodeled bone and cartilage-containing vertebrate skeletons may be regulated by the enzymatic control of polyphosphate synthesis and degradation.

## Methods

### Back scattering electron (BSE) imaging and energy dispersive X-ray spectroscopy (EDX)

PolyP solubility is lower in acetone than in aqueous solutions [75]. To reduce the polyP dissolution during sample preparation, we used acetone-dehydrated, undecalcified, 9-month-old excised guinea pig tibiae cut coronally and embedded them in Spurr® resin (Electron Microscopy Sciences, Hatfield, PA). All sections were dry-polished with 4000-grit paper. We generated BSE images in an ESEM (XL30, FEI Company, Hillsboro, OR, 20 kV, 60 µA) and collected qualitative EDX spectra (Phoenix EDX detector, EDAX, Mahwah, NJ).

### Identification of polyP in skeletal tissue by fluorescence microscopy

We used fluorescence microscopy to identify polyPs via the specific fluorescence of the DAPI-polyP complex, which differs markedly from the fluorescence of the DAPI-DNA complex. DAPI has been used to identify polyPs in phytology, microbiology, and molecular biology because the emission wavelength for the DAPI-polyP complex is shifted towards 520 nm, in contrast to the 460–465 nm emission fluorescence of the DAPI-DNA complex [23], [26], [62], [76].

We demineralized 3-month-old murine vertebral bodies (0.3 M EDTA, pH 7.4, 4°C, 10 days), embedded them in paraffin wax, dry sectioned them to 5–10 microns (Reichurt-Jung BioCut 2030, Leica Microsystems Canada Inc., Richmond Hill, ON) and mounted them on slides. For removing bone mineral, we preferred the cold EDTA processing method over acid because hot and/or acidic conditions are known to accelerate the kinetics of polyP hydrolytic degradation [77]. Some of the cold sections were stained with DiI (2% in ethanol, 30 min, D-282, Invitrogen Canada Inc., Burlington, ON) to identify the plasma membranes. DiI was used to navigate sections excited by a 488 nm laser. Without dewaxing, all sections were exposed to DAPI (50 µg/mL in 0.2 M TRIS, pH 9, 5 min, Pierce Biotechnology, Inc., Rockford, IL) for 2 minutes, then rinsed with TRIS (0.2 M, pH 9) solution.

**Figure 10:**
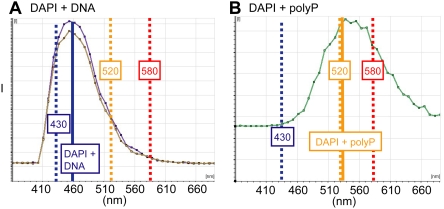
Emission spectra of DAPI-DNA and DAPI-polyP. (A) Emission spectra of DAPI-DNA obtained from murine brain section. Note position of maximum intensity at 460 nm, intensity at 430 nm, and intensity at 520 nm. The intensity at 430 nm is used as a proxy for the contribution of the DAPI-DNA curve to the convoluted DAPI-DNA-polyP spectra. (B) Emission spectrum of DAPI-polyP obtained from synthetic polyP. Note position of maximum intensity near 520 nm and minimal intensity at 430 nm. The intensity at 520 nm is used as a proxy for the contribution of the polyP to the convoluted DAPI-DNA-polyP spectra in bone sections. Fluorescence above 580 nm is exclusively due to DAPI-polyP emission and was used for imaging purposes in Figures 2 and 5.

The labeled sections were excited with a multiphoton laser (Milennia XS – Tsunami, SpectraPhysics, CA) at ∼780 nm, in accordance with the suggested DAPI excitation wavelengths by Neu *et al.* [78]). The multiphoton laser was directly coupled to a Leica SP2 confocal scanning microscope system. Wavelength scans (xyλ) were acquired in 20 nm bins (400–700 nm) that allowed spectral analysis of the emission spectra. The DAPI-DNA complex emission peak was detected and identified based on its 460–465 nm peak emission, and the DAPI-polyP complex peak based on its 520–580 nm emission (Figure 10, solid lines). We assumed that emissions at 580 nm represented DAPI-polyP emissions minimally convoluted with DAPI-DNA ones (Figure 10, red dashed line); we therefore used the 580 nm emissions to represent DAPI-polyP emission regions (for example, in Figures 2, 5). The DAPI-DNA emissions for Figure 10A were collected from a murine brain section, while Figure 10B depicts the emission spectra from a solution of 10 µg/mL sodium polyphosphate (Type 28, Sigma-Aldrich) and 10 µg/mL DAPI in TRIS (0.2 M, pH 9). Spectral scans were analyzed with Leica LCS or Leica Lite® software. Mathematical subtraction or addition of spectral emission curves (Figure 6C, dashed lines) was re-normalized so that the peak intensity equaled 0.5 before plotting.

### Tartrate-resistant acid phosphatase staining

We used a kit (Sigma-Aldrich # 181-A) to stain for tartrate-resistant acid phosphatase (TRAP) as a marker for osteoclasts [64]. Each decalcified, dewaxed section was incubated for 2 hours with a mixture of naphthol AS biphosphoric acid (12.5 mg/mL), tartrate solution (0.67 M final conc., pH = 5), acetate solution (2.5 M final conc., pH = 5), and fast red TR salt (0.1 g/10 mL). The section was then counterstained with haematoxylin.

### Toluidine blue staining

Demineralized (0.3 M EDTA, pH 7.4, 4°C, 10 days), 3-month-old murine vertebral bodies were embedded in paraffin wax, dry sectioned to 5–10 microns (Reichurt-Jung BioCut 2030) and mounted on slides. We stained sections with toluidine blue (0.01% (Sigma-Aldrich), 0.01 M acetate, pH 4, filtered) for up to 10 minutes [50] and imaged them using a Retiga 1300 Camera with QImaging (image capture software suite 2.0), a Zeiss microscope with a Dell Optiplex GX240, and a resolution set at 1024×768.

### Reduction of polyphosphate content in the growth plate with alkaline phosphatase

We prepared sections of growth plates from wild type murine vertebral bodies/tibial plateaus as described for polyphosphate detection by fluorescence microscopy. Immediately before staining and imaging, we cut 5–10 micron sections and exposed them to either an ALP-free buffer solution serving as control (10 mM TRIS, pH 8.2, 50 mM KCl, 1 mM MgCl_2_, 0.1 mM ZnCl_2_, 37°C; Sigma-Aldrich, 0.2 M, pH 9) or to an intestinal ALP solution (10 U/mL ALP, bovine calf intestine, P7923-2KU, Sigma-Aldrich, dispersed in buffer) for 5 minutes. We applied ALP to accelerate the hydrolytic degradation of polyP within the growth plate section. All sections were subsequently exposed to DAPI (50 µg/mL) for 2 minutes. After a rinse with TRIS solution, the sections underwent a spectral scan as described for polyP detection by fluorescence microscopy.

In order to compare the emission spectra, we used data collected with the Leica LCS software to determine the position of maximum intensity, the spectrum intensities at 430 and 520 nm, and the full width at the half-maximum intensity (FWHM) of the intensity-wavelength curves. The emission spectra for the DAPI-DNA and DAPI-polyP curves are convoluted; there is minimal overlap in the two emission spectra at 430 and >500 nm (Figure 10). In order to quantify the emission data, the intensity at 430 nm was used as an index of the DAPI-DNA emission (Figure 10, blue dashed line), while the intensity at 520 nm represented the DAPI-polyP component (Figure 10, yellow dashed line). The ratio of emission intensities at 520 nm and 430 nm is proposed to represent the relative intensity of DAPI-polyP in the convoluted emission spectra; when calculating this 520∶430 nm ratio, the intensity values were first subtracted from the individual background intensity. We have assumed that ALP selectively decreases the DAPI-polyP emission spectrum by accelerating the hydrolytic degradation of the polyP while leaving the DNA unaffected.

We propose that the wavelength position of maximum intensity and the full width at half max (FWHM) also provide information about the contribution of the DAPI-polyP emission spectrum to the total DAPI-DNA/DAPI-polyP spectrum. A reduction of the DAPI-polyP component of the convoluted spectra is assumed to result in a shift of the position of maximum intensity to shorter wavelengths as well as a reduction in the FWHM of the total emission curve. In some of the images (Figures 2, 5), we show that the DAPI fluorescence at 580 nm indicates the polyP signal; the use of 580 nm as an index is based on the fact that we found nearly all of the DAPI-DNA emission spectrum to be below 580 nm while more than a third of the DAPI-polyP profile remained at wavelengths above 580 nm (Figure 6).

### Tissue-nonspecific alkaline phosphatase enzymatic assay

We mixed bovine kidney alkaline phosphatase (a tissue-nonspecific alkaline phosphatase [54]) (Calzyme Laboratories, Inc. San Luis Obispo, CA) at a final concentration of 7 units/mL in 0.2 M TRIS (Sigma-Aldrich, pH 9) with sodium polyP (Type 28, Sigma-Aldrich in 0.2 M TRIS, pH 9, final concentration 30 mM equivalent Pi) and incubated the mixture at 37°C. TNAP-free, polyP solutions served as the control. Aliquots taken at each time point were mixed with an equal volume of 0°C, 1 M NaOH solution. We used polyacrylamide gel electrophoresis (PAGE) to separate the phosphate species in 25 µL samples of a mix containing a 1∶1.5 ratio of denatured aliquots (initial polyP solution, TNAP time points, or the TNAP-free final time point serving as a control) to sample buffer (90 mM TRIS, 90 mM borate, 2.7 mM EDTA, 0.05% bromophenol blue, pH 9, 20% sucrose).

Vertical slab gels (200×200×0.5 mm) were cast with 16% polyacrylamide (19∶1 bisacrylamide∶acrylamide ratio, 7 M urea and 1×TBE (90 mM Tris, 90 mM borate and 2.7 mM EDTA, pH 8.3 [25], [79])). The standard lane contained Pi (20 nmoles) and pyrophosphate (P_2_O_7_^4−^, 20 nmoles) ladders. The polyphosphate/phosphate products were separated at approximately 15 V(cm)^−1^ with internal water cooling for 1.5–2 hours, then visualized with methyl green after *in situ* hydrolytic degradation [80].

### Adsorption of polyphosphate to hydroxyapatite

In order to determine whether longer chain polyP molecules could adsorb to HAP crystals in solution, we adapted the sequential method from Michelmore *et al.* [81] with a background electrolyte of 0.15 M NaCl pre-saturated with synthetic HAP. Synthetic HAP (Sigma-Aldrich, Milwaukee, WI) was used as a proxy for the apatite mineral in bone.

In this method, we sequentially added phosphate, as either Pi (0.01 M equivalent phosphate (Na_2_HPO_4_)) or polyP (Na(PO_3_)_n_, Type 28 (average polyP chain length = 25 Pi, Sigma-Aldrich, both dissolved in 0.15 M NaCl pre-saturated with HAP)), into HAP-presaturated solutions that were either unseeded (contained no HAP mineral seeds) or seeded (contained 2 g/L HAP aged mineral seed in suspension). The amount of Pi or polyP added was normalized to moles of equivalent P by calculating the total moles of Pi added as Pi or polyP. We collected an aliquot (0.5% of the initial volume) from the test solution before each incremental delivery (0.5% of the initial test volume) of Pi or sodium polyP.

Aliquots were centrifuged (10,000 rpm, 5 min) and the supernatant analyzed for free calcium with ortho-cresolphthalein [82] and free Pi with vanadomolybdate [83] by colourimetry. We mixed equivalent volumes of the supernatant samples with 0.1 M HCl in microtubes and heated them to 100°C overnight to completely hydrolytically degrade the polyP before measuring the total (free+sequestered) calcium and total (ortho+polyphosphate) Pi concentrations [84]. Acidified solutions were neutralized with 0.1 M NaOH before colourimetric analysis of total calcium concentrations to maintain a basic pH for the assay.

We concluded that an adsorption of Pi or polyP to the HAP seed occurred if the total Pi concentration was reduced in the supernatant solutions that contained HAP seed compared to those that did not contain it for equivalent phosphate species additions.

### Measurement of HAP-relative saturation

The relative supersaturation with respect to HAP (σ_HAP_) is a function of the thermodynamic solubility product (K_sp_) and the ionic activity product (IAP) at a given temperature:
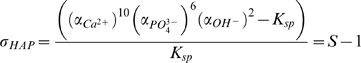
where S is defined as saturation [85], α is the ion activity, and σ is defined as relative saturation. σ is an indicator of the thermodynamic force driving towards dissolution or growth of a specific phase in solution. A relative saturation of zero (σ = 0) represents an ideal system in which the solution is at thermodynamic equilibrium with the salt. Supersaturated systems (σ>0) favor nucleation and growth, while undersaturated systems (σ<0) progress towards mineral dissolution.

Ionic activities (α), representing the chemical potential of an ion in solution, are a function of the ion molal (mol/kg solvent) concentration and the activity coefficient (γ), which in turn is a function of the ionic strength and composition of the solution. The ionic activity is an indicator of the “free” concentration of an ion in a given solution. We calculated the relative saturation of HAP using the free calcium, Pi, and pH data [85], [86] for the HAP-seeded solutions studied in the adsorption experiment.

## Supporting Information

Figure S1 Densitometry of Figure 7 – TNAP cleaves Pi from polyP. Separation of synthetic polyP exposed to TNAP by polyacrylamide gel electrophoresis (PAGE). TNAP exposure times were 0.5, 1, 2.5, 5, 10, 15, 20, and 30 minutes. Plot of the signal intensity from polyP (filled circles), tripolyphosphate ((P_3_O_10_)^5−^, open circles), and orthophosphate (Pi, filled triangles) with increasing exposure to TNAP. Synthetic polyP contains a range of polyP species, from tripolyphosphate (3 condensed Pi units) to polyP chains longer than 28 Pi units. With increasing exposure to TNAP, the PAGE shows a trend of increasing Pi and decreasing polyP concentration.
(0.36 MB TIF)

Figure S2 Orthophosphate (Pi) and polyphosphate (polyP) adsorption data to synthetic hydroxyapatite (HAP) in 0.15 M NaCl saturated with respect to HAP. After complete hydrolytic degradation of the supernatant in hot, acidic conditions, the measured free Pi concentration is assumed to represent the total Pi concentration. The free calcium concentration after hydrolytic degradation is also assumed to represent the total calcium concentration (unseeded: without HAP crystals; seeded: with HAP crystals). The data will be discussed with respect to the Pi adsorption experiment, followed by the polyP adsorption experiment. *Pi adsorption data:* Free, unseeded (A, open circles) and seeded (A, open triangles) Pi concentrations with increasing Pi addition were similar, indicating no measurable adsorption of Pi to HAP. The same increase in total Pi with Pi addition was also observed for the unseeded (B, open circles) and HAP-seeded (B, open triangles) adsorption experiments. The free calcium concentrations (C, open circles), total calcium concentrations (D, open circles), and pH (E, filled circles) in the unseeded Pi adsorption experiment remained unchanged. For the HAP-seeded experiment, the free calcium concentration decreased slightly with increasing Pi addition (C, open triangles), as did the pH, which is also affected by the buffering capacity of Pi (F, closed circles). This result suggests some HAP seed growth as a consequence of Pi addition to the HAP-saturated system. Increasing the Pi concentration would be expected to increase the HAP saturation, favouring HAP crystal growth. *PolyP adsorption data:* The free Pi concentration in the polyP adsorption experiment increased slightly with time for the unseeded (A, filled circles) and HAP-seeded (B, filled triangles) experiments. The addition of polyPs to a solution saturated with HAP is not expected to change the free Pi concentration appreciably. In an aqueous solution, however, thermodynamics predicts the spontaneous hydrolytic degradation of polyP into Pi ions. Some polyP hydrolytic degradation in our experimental conditions may explain the increase in Pi concentration in the polyP adsorption experiment (A, filled symbols) and decrease in pH (E, open circles). Hydrolytic degradation of polyP in the supernatant showed a lower total Pi concentration for the HAP-seeded experiment (B, filled triangles) than the unseeded (B, filled circle) experiment, indicating adsorption of polyP to HAP seed. Free calcium concentrations in the supernatant of the polyP addition experiment decreased to zero for the unseeded system (C, filled circles) and to lower concentrations than those to which Pi was added for the HAP-seeded system (C, filled triangles). We assumed that the sequestration of calcium by polyP was stronger than the indicating calcium-orthocresolphthalein complex; therefore no free calcium was detected by colourimetry. For the unseeded system, the total calcium concentration (D, filled circles) remained unchanged with increasing polyP. Interestingly, the total calcium concentration (D, filled triangles) and pH (F, open circles) both increased with increasing polyP addition in the HAP-seeded system, suggesting dissolution of the HAP seed during the adsorption experiment.
(0.88 MB TIF)
