# Supplementary figures and images for: Control of Vertebrate Skeletal Mineralization by Polyphosphates
Source: PLoS One. 2009 May 20;4(5):e5634. doi: 10.1371/journal.pone.0005634 (PMC2683572; doi:10.1371/journal.pone.0005634)

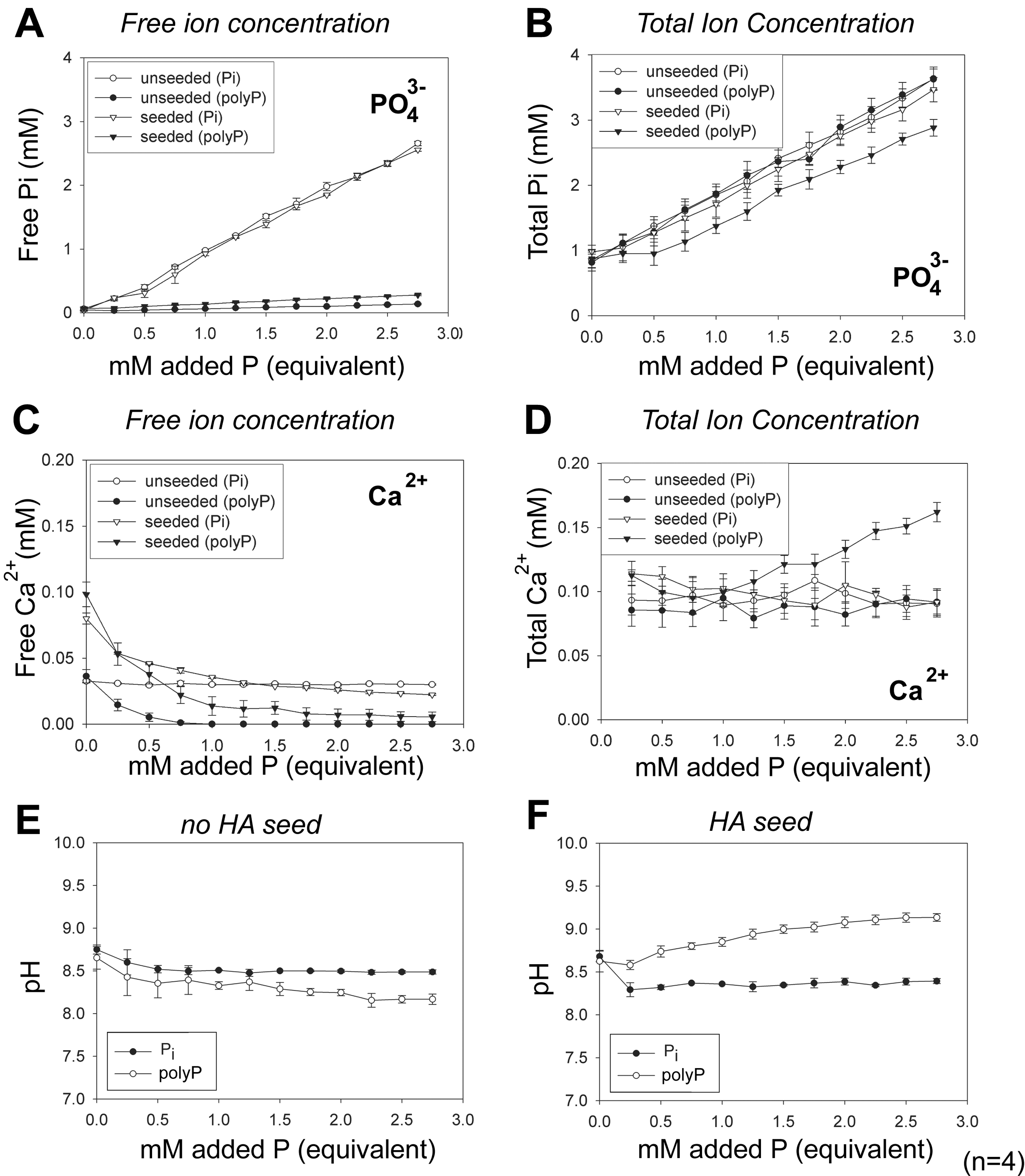

Supplement: Figure S2 — Orthophosphate (Pi) and polyphosphate (polyP) adsorption data to synthetic hydroxyapatite (HAP) in 0.15 M NaCl saturated with respect to HAP. After complete hydrolytic degradation of the supernatant in hot, acidic conditions, the measured free Pi concentration is assumed to represent the total Pi concentration. The free calcium concentration after hydrolytic degradation is also assumed to represent the total calcium concentration (unseeded: without HAP crystals; seeded: with HAP crystals). The data will be discussed with respect to the Pi adsorption experiment, followed by the polyP adsorption experiment. Pi adsorption data: Free, unseeded (A, open circles) and seeded (A, open triangles) Pi concentrations with increasing Pi addition were similar, indicating no measurable adsorption of Pi to HAP. The same increase in total Pi with Pi addition was also observed for the unseeded (B, open circles) and HAP-seeded (B, open triangles) adsorption experiments. The free calcium concentrations (C, open circles), total calcium concentrations (D, open circles), and pH (E, filled circles) in the unseeded Pi adsorption experiment remained unchanged. For the HAP-seeded experiment, the free calcium concentration decreased slightly with increasing Pi addition (C, open triangles), as did the pH, which is also affected by the buffering capacity of Pi (F, closed circles). This result suggests some HAP seed growth as a consequence of Pi addition to the HAP-saturated system. Increasing the Pi concentration would be expected to increase the HAP saturation, favouring HAP crystal growth. PolyP adsorption data: The free Pi concentration in the polyP adsorption experiment increased slightly with time for the unseeded (A, filled circles) and HAP-seeded (B, filled triangles) experiments. The addition of polyPs to a solution saturated with HAP is not expected to change the free Pi concentration appreciably. In an aqueous solution, however, thermodynamics predicts the spontaneous hydrolytic degrad [file pone.0005634.s002.tif]
